# Body-Esteem, Self-Esteem and Loneliness among Social Media Young Users

**DOI:** 10.3390/ijerph19095064

**Published:** 2022-04-21

**Authors:** Lavinia Maria Pop, Magdalena Iorga, Raluca Iurcov

**Affiliations:** 1 Faculty of Psychology and Education Sciences, “Alexandru Ioan Cuza” University of Iasi, 700554 Iasi, Romania; lavinia-maria.pop@umfiasi.ro; 2 Behavioral Sciences Department, “Grigore T. Popa” University of Medicine and Pharmacy, 700115 Iasi, Romania; 3 Dentistry Department, Faculty of Medicine, University of Oradea, 410073 Oradea, Romania; riurcov@uoradea.ro

**Keywords:** body image, body mass index, body esteem, loneliness, self-esteem, young adults, medical students, social network, health

## Abstract

The use of social networking sites for socializing, having fun, solving academic tasks or even getting counselling for health-related problems is now inevitable. Methods: A total of 427 medical students, who are users of social media sites, were included in the research. Data about socio-demographic, anthropometric, and self-rated items regarding satisfaction with physical and mental health were collected. Three psychological tools were also used to measure self-esteem (*Rosenberg Self-Esteem Scale)*, body-esteem (*Body Esteem Scale for Adolescents and Adults*) and loneliness (*UCLA Loneliness Scale)*. Collected data were analyzed using SPSS version 23. Results: Students use these networks for socialization (49.0%), entertainment (31.1%) and academic tasks (19.9%), spending 3.38 ± 0.80 h per day on SNSs. Less than half of them (47.5%) compared themselves to other SNS profiles. The use of Snapchat was found to be strongly positively correlated with self-esteem, and weight status was negatively correlated with the use of TikTok. More than three-quarters declared that they exercised to lose weight or to prevent weight gain. Participants were found to have a high level of body esteem. Almost half of the students proved to have a moderate to a high level of loneliness. Age and gender were found to be important: the younger the user, the higher the scores for loneliness and feeling depressed, and the greater the number of hours on SNSs. The total score for self-esteem was significantly higher in men than in women, and male students appreciated themselves as being in a better state of mental health than women. Conclusions: The results prove a relationship between the use of SNSs and the presence of loneliness, self-esteem and body-esteem, with gender differences. However, the use of SNSs should not be neglected in clinical settings, and are a good means of reaching patients and providing medical and psychological intervention.

## 1. Introduction

In a world that is constantly connected, in which individuals from different cultures and who speak different languages can relate and find common goals of communication, identifying the degree of loneliness among these individuals, especially for those at an age when social relationships are strongly stimulated, seems like a paradox.

One area of Internet usage that has become increasingly popular is the use of social networking sites (SNSs). Data provided by Social Networking Fact Sheets, in 2015, showed that 74% of online adults were using SNSs [1]. A growing body of scientific literature has explored the association between general social networking site (SNS) use and loneliness, although results have not clarified whether SNS use increases or decreases loneliness. Therefore, SNS use has been related not only to greater social connectedness and wellbeing [2], but also to increased loneliness [3]. 

In contemporary society, numerous young people move away from their personal networks for extended periods to achieve professional and/or educational goals. This separation can often lead to feelings of loneliness, which can be stressful for the individual [4]. 

Loneliness is a subjective psychological state, and has been associated with objective social isolation, depression, introversion or poor social skills. Loneliness is defined as a unique condition in which an individual perceives himself or herself to be socially isolated even when among other people [5]. It has been found to steadily increase with age and, almost consistently, to be strongly associated with poor health conditions and unfavorable behaviors across all ages. It has also been found to be far more prevalent in today’s society than in previous generations [6]. Loneliness has been shown to be most prevalent in different social categories: young adults (aged 18–29 years); older adults; people with a physical or mental health condition; people with low income; and people with different marital statuses (single, separated, widowed, or divorced [7]).

Loneliness has been found to have a wide range of negative impacts on an individual’s psychological and physical health [7], and is often wrongly conflated with social isolation; research identifies that loneliness can occur in the presence of others.

Fox [8] found that loneliness was a predictor for social media use and an aggravating factor for those at risk of health, especially regarding body image and eating disorders such as anorexia or bulimia. Çivitci and Çivitci [9] found that loneliness was negatively associated with life satisfaction and self-esteem. Çiçek [10] also showed that self-esteem had a mediating role in the association between loneliness and psychological well-being in young adults.

Neff and Vonk [11] identified that self-esteem (an evaluation of adequacy that is derived from positive self-appraisals and favorable comparisons with others) was consistently correlated with higher body satisfaction. According to Albertson et al. [12], the prevalence of body dissatisfaction among teenagers and young women in Western European countries is widespread, with the majority reporting negative thoughts and feelings about their weight and body shape. 

Hardit and Hannum [13] showed that the main predictors of body dissatisfaction were parental influence, peer influence, and mass media. The authors claimed the last factor was the most significant. Zinovyeva et al. [14] identified that young people with body dissatisfaction tended to narrow their social contacts or even isolated themselves.

Shwartz [15] identified that self-esteem was negatively related to the use of SNSs, suggesting that the more frequently people went on SNSs, the lower their self-esteem. Chen and Qin [16] concluded that decreasing loneliness and increasing self-esteem should be applied in interventions to reduce social anxiety among teenagers. 

People with low self-esteem were proven to be more prone to using SNSs. The consequences are self-isolation; neglecting of personal relationships; increasing risk of psychological problems, such as depression, loneliness, dietary and sleep disturbances; and dissatisfaction with personal and social relationships [17].

The impact of SNSs on mental health has been frequently described, and sometimes reflected in opposing findings. For example, O’Keeffe and Clarke-Pearson [18] pointed to the “Facebook depression”, whereas Jelenchick [19] showed that the use of SNSs in clinical settings had an extremely important positive impact on patients’ recovery, especially in mental health problems, on the condition that the person was not addicted to SNSs.

The aims of the present study were to assess the presence of body-image satisfaction, self-esteem, and loneliness among the young users of SNSs and to identify the relationship between them. Comparative analysis between different categories was also assessed in order draw conclusions considering gender, marital status, financial income, age, or dieting.

## 2. Materials and Methods

### 2.1. Participants

**Figure 1:**
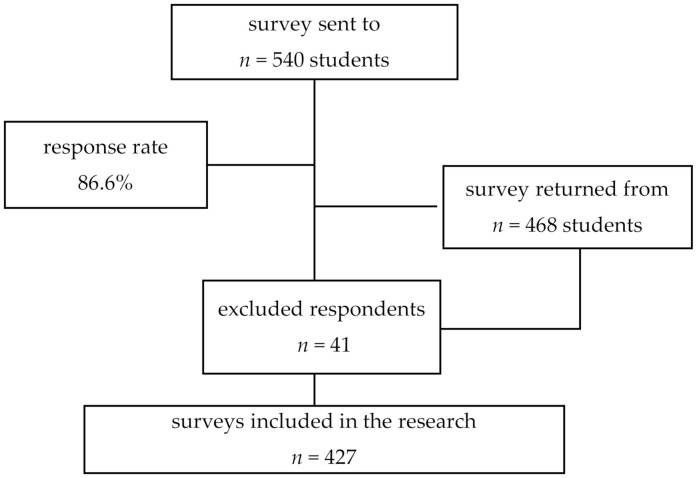
Study profile.

The study was conducted in September–November 2021 in two medical universities in Romania. The questionnaires were distributed online to a total of 540 medical students enrolled across all academic years of study. Participants were informed about the purpose of the study, about the fact that they could withdraw from the study whenever they wanted, without consequences, and about the confidentiality of data. No incentive was given to respondents. The inclusion criteria were that participants had to be students enrolled in one of the universities, and filling out the questionnaire before the deadline. The exclusion criteria were questionnaires incomplete or submitted after the deadline. Finally, 427 questionnaires were included in the research. Figure 1 provides details on the response rate. 

### 2.2. Data Collection

The online questionnaire was created using the Google Forms application (Alphabet, Mountain View, CA, USA) and was developed to obtain detailed information about students’ perceptions of body image and the influence of social media, and to self-evaluate body image, appearance, self-esteem and level of loneliness. 

The first part of the questionnaire gathered socio-demographic, academic and anthropometric information (such as age, gender, marital status, home environment, year of study, members of the household, monthly financial income, weight status and self-evaluation of weight status).

The second part questioned respondents’ satisfaction about body image and physical, mental and social health; the level of satisfaction regarding perceived emotional support from family and friends; and self-assessments of depression and stress levels. Self-assessed items were specially constructed for this questionnaire after a literature review. Responses were rated on a 5-point Likert scale, except for the item on body image satisfaction, which was assessed using a 10-point Likert scale.

The third part of the survey collected information about the use of social media (such as Facebook, Instagram, WhatsApp and YouTube), the main reason for using these applications, the average time spent on these networks a day, and the level of interest shown by users in the number of positive or negative reviews they received for the content posted.

The final part of the questionnaire included three psychological tools in order to assess appearance and body image, self-esteem and loneliness:

- *Body Esteem Scale for Adolescents and Adults* [20] was developed by Mendelson et al. in 2001 and comprises 23 items. This instrument is a self-report tool that includes 3 subscales: private general feelings about appearance, weight satisfaction, and evaluations attributed to others about one’s body and appearance. The respondents needed to indicate their degree of agreement on a 5-point Likert scale ranging from 0 (never) to 4 (always). The scores for each subscale and the total were computed by simple addition. A higher BES score indicates a higher level of body esteem.
- *Rosenberg Self-Esteem Scale* [21] consists of 10 items and is a self-report instrument for evaluating individual self-esteem. RSES was scored using four response choices, ranging from strongly agree to strongly disagree.
- *UCLA Loneliness Scale (ULS-8)* contains the 8 items selected from the revised UCLA Loneliness Scale of Hays and DiMatteo [22]. The answers are scored on a 4-point Likert scale from 1 (never) to 4 (always). The ULS-8 is a commonly used tool developed to measure one’s subjective feelings of loneliness, and feelings of social isolation.

### 2.3. Statistical Analysis

The statistical analyses were performed using IBM Statistical Package for Social Sciences (SPSS) for Windows, version 24 (SPSS Inc., Chicago, IL, USA). Results for descriptive statistics are expressed as means and standard deviations (SD). 

Body mass index (BMI) was computed and used to classify the participants into normal, underweight, and overweight/obese groups according to WHO guidelines, using standards for the European population: a BMI < 18.5 kg/m^2^ was categorized as underweight, 18.5–24.9 kg/m^2^ as normal weight, 25.0–29.9 kg/m^2^ as pre-obese, 30–34.9 kg/m^2^ as obese class I, 35.0–39.9 kg/m^2^ as obese class II, and ≥40 kg/m^2^ as obese class III [23].

The normality of data distribution was tested -using the Kolmogorov–Smirnoff test. Given the fact that all data are not normally distributed, a bivariate analysis was performed and non-parametric tests were applied. To assess comparative results considering gender, origin environment and use of social media, the Mann–Whitney test was performed. 

Comparative results considering marital status, study university, type of housing, members of the household, average monthly financial income, and weight status were assessed using the Kruskal–Wallis H test to determine if there were statistically significant differences between more than two groups of an independent variable on a continuous or ordinal dependent variable. 

Multiple linear regression analyses were performed to investigate possible effects of different variables on the BESAA, RSES and ULS-8 scores of students. Only those variables that attained significance in the correlation analysis were considered in the regression analyses.

The Spearman correlation was used to test the relationship between variables. A *p*-value < 0.05 was considered statistically significant. 

### 2.4. Ethical Approval

The study was approved by the Ethical Committee of the Faculty of Medicine at the University of Oradea, Romania, with the registration No. CFMF01/30.04.2021.

## 3. Results

### 3.1. Socio-Demographic, Anthropometric and Academic Data

Sociodemographic data were gathered, together with financial status, environment characteristics, and household-related information. 

The students were enrolled in all years of study (1 to 6), and aged M = 21.62 ± 2.37 (with a minimum of 18 and a maximum of 30 years old). Most students were female (82.40%, *n* = 352) and more than half came from urban areas (71.70%, *n* = 306).

Body mass index (BMI) was calculated, conforming to WHO guidelines, and we identified that 63% of students had a normal weight. 

Students were asked if they practiced physical activity in order to maintain their weight or to lose weight. A large majority of students declared that they exercised to lose weight or to prevent weight gain (80.6%, *n* = 344). Similarly, students were asked to self-rate their level of satisfaction with their weight status. Detailed information about socio-demographic and anthropometric data is presented in Table 1. 

### 3.2. Satisfaction with Health-Status and Relationship with Family and Friends 

The results indicated that most of the students had to rate themselves on a scale from 1 (extremely unsatisfied) to 10 (extremely satisfied) regarding their body image (M = 7.21 ± 1.87). Respondents were also asked to self-rate their level of depression and stress, and to evaluate the emotional support offered by family and friends. The results showed that students did not feel depressed (M = 2.42 ± 1.14), but instead felt a higher level of stress (M = 3.43 ± 1.13). Other detailed results are presented in Table 2. 

### 3.3. Use of Social Media

On average, students spent 3.38 ± 0.80 h on social media. Respondents were asked to indicate which SNS they usually used. The results showed that, of the six social networks, YouTube (98.8%, *n* = 422), WhatsApp (98.4%, *n* = 420), Facebook (93.2%, *n* = 398) and Instagram (88.1%, *n* = 376) were the most frequently used, followed by Snapchat (48.5%, *n* = 207) and TikTok (47.3%, *n* = 202). 

The main reasons why students used these networks were primarily socialization (49.0%, *n* = 209) and entertainment (31.1%, *n* = 133), and they were less used for solving academic tasks (19.9%, N = 85). 

Fewer than half of the students (47.5%, *n* = 203) compared themselves to others when viewing different profiles, and they were quite uninterested in the number of likes (M = 2.70 ± 3.00) or dislikes (M = 2.19 ± 2.00) for their posts. 

### 3.4. Body Esteem Scale for Adolescents and Adults (BESAA)

The Cronbach alpha score was 0.94 for the total scale, 0.92 for BES-Appearance, 0.92 for BES-Weight and 0.68 for BES-Attribution. For BESSA subscales we obtained the following results:

- Appearance (BES-Appearance)—M = 33.46 ± 8.55,
- Weight Concern (BES-Weight)—M = 25.92 ± 7.97,
- Attribution (BES-Attribution)—M = 23.13 ± 3.27.

The results proved that respondents had a high level of body esteem. The total BES score was, on average, M = 82.52 ± 17.85, and scores ranged from 38 (0.7%, N = 3) to 117 (0.5%, N = 2). No significant differences in terms of participants’ gender were found. Table 3 presents the results for the subscales. 

A Mann–Whitney U test was conducted to determine if there were differences in BESAA total score between participants who exercised to lose weight or to prevent weight gain and those who did not. The BESSA total score was statistically significantly higher in students who did not perform exercise (Mdn = 94.00) than in students who practiced physical activity (Mdn = 81.00), *U* = 8962.0, *z* = −5.267, *p* < 0.01). 

The comparative analysis also showed that there were significant differences in the BESAA score for the variable that refers to the self-assessment of satisfaction with body image (*p* < 0.001, *U* = 2010.50, *z* = −11.367); that is, those who self-rated as having lower satisfaction with their own body (Mdn = 58.00) obtained a lower score in the body esteem scale compared to those who estimated a higher satisfaction with their own body (between points 6 and 10 on the Likert scale) (Mdn = 88.50).

A Kruskal–Wallis H test was conducted to determine if there were differences in total BESAA score between groups that differed in their nutritional status: the “underweight” (*n* = 48), “normal weight” (*n* = 271), “overweight” (*n* = 86), “obese class I” (*n* = 19), and “obese class II” (*n* = 3) groups. The mean ranks of BESAA scores were statistically significantly different between groups, χ^2^(4) = 67.697, *p* < 0.001. The analysis of the data showed that there were differences in total BESAA score between groups that differed in their weight status: the “underweight” (*n* = 48), “normal weight” (*n* = 271), “overweight” (*n* = 86), “obese class I” (*n* = 19), and “obese class II” (*n* = 3) groups. Median BESSA scores were statistically significantly different between the different levels of weight status group, χ^2^(4) = 67.697, *p* < 0.001. 

A post hoc Mann–Whitney U test was conducted to determine which groups were different from which other groups. This post hoc analysis revealed statistically significant differences in BESAA score between the normal weight (Mdn = 88.00) and class I obesity groups (Mdn = 55.00) (*p* < 0.001, *U* = 805.00, *z* = −5.009), and overweight (Mdn = 73.00) and class II obesity groups (Mdn = 46.00) (*p* = 0.019, *U* = 26.00, *z* = −2.343). Similarly, the median BESAA scores for underweight (Mdn = 93.50) and overweight groups (Mdn = 73.00) (*p* < 0.001, *U* = 992.50, *z* = −4.974), and normal weight (Mdn = 88) and overweight groups (Mdn = 73) (*p* < 0.001, *U* = 6777.50, *z* = −5.848), were statistically significantly different.

### 3.5. Rosenberg Self-Esteem Scale

For the present study, the Cronbach alpha score was 0.885. 

Global self-esteem scores as measured with the RSES ranged from 13 to 27 (M = 22.30 ± 2.48). 

A Mann–Whitney U test was conducted to determine if there were differences in RSES total score between males and females. We identified that the RSES total score was statistically significantly higher in male (Mdn = 21.00) than in female students (Mdn = 20.00), *U* = 10740.5, *z* = −2.557, *p* = 0.01). 

The comparative analysis also showed that there were significant differences in the RSES score for the variable that refers to the self-assessment of satisfaction with body image (*p* < 0.003, *U* = 10075.00, *z* = −2.990); that is, that those who estimated lower satisfaction with their own body (Mdn = 20.00) obtained a lower score of self-esteem compared to those who estimated a higher satisfaction with their own body (Mdn = 21.00).

### 3.6. Loneliness Scale (ULS-8 Short Form)

The scale had good internal consistency, as determined by a Cronbach’s alpha of 0.882. 

The total score for the Loneliness scale was M = 15.78 ± 4.58, and scores ranged from 8 (3.3%, *n* = 14) to 30 (0.2%, *n* = 1). Almost half of the students (45.2%, *n* = 1933) were found to have a moderate to high level of loneliness. The detailed results are presented in Table 4. 

The Kruskal–Wallis H test revealed that there were statistically significant differences in the total Loneliness scale score between groups with different marital status: the “single” (*n* = 200), “in a relationship” (*n* = 216) and “married” (*n* = 11) groups; χ^2^(2) = 9.046, *p* = 0.011. The Mann–Whitney U post hoc analysis (*p* = 0.009, *U* = 18398.50, *z* = −2.619) showed that people who were in a relationship (Mdn = 15.00) had a lower level of loneliness than single people (Mdn = 16.00). No gender differences were identified.

Comparative analysis showed that men (Mdn = 2.00) felt less depressed (*p* < 0.001, *U* = 9719.50, *z* = −3.708) and less stressed (*p* = 0.002, *U* = 10244.50, *z* = −3.150, Mdn = 3) than women (Mdn = 3, Mdn = 4). Furthermore, the results of the Mann–Whitney test (*U* = 11013.00, *z* = −2.362, *p* = 0.018) showed that men (Mdn = 4) perceived themselves to be in a better state of mental health than women (Mdn = 3).

Comparative analysis showed that students who exercised in order to lose weight (*p* < 0.001, *U* = 8962.00, *z* = −5.267) had a lower body esteem score (Mdn = 81) than those who did not exercise (Mdn = 94). Similarly, students who exercised (*p* < 0.001, *U* = 11017.50, *z* = −3.405) had a lower score on the physical health satisfaction scale (Mdn = 3) than those who did not exercise in order to lose weight (Mdn = 4).

Our results showed that there was a positive correlation between the total score of BESAA and the use of social networks. Thus, we identified that the more time the students spend on TikTok (r = 0.15, *p* = 0.002) or Snapchat (r = 0.12, *p* = 0.01), the greater their body esteem. Similarly, the use of Snapchat is strongly positively correlated with the RSES score, so the more the students spent time on Snapchat, the higher their self-esteem (r = 0.12, *p* = 0.008). 

A strong negative correlation was identified between the total score of BESAA and weight status, meaning the higher the student’s BMI, the lower the body esteem score (r = −0.37, *p* < 0.001). Weight status was negatively correlated with the use of TikTok; thus, the higher the BMI, the less students will use TikTok (r = −0.11, *p* = 0.02).

Age was found to be an important factor. The older the students, the lower the level of loneliness (r = −0.11, *p* = 0.01). Similarly, the older the students, the lower the level of depression (r = 0.10, *p* = 0.02). Moreover, the older the students, the lower the number of hours a day spent on social networks (r = −0.24, *p* < 0.001).

A positive correlation was identified between the total score of RSES and financial income; that is, the higher the income, the higher the self-esteem (r = 0.11, *p* = 0.01). Additionally, the results show that, the more income students have per month, the more likely they are to use YouTube more often (r = 0.09, *p* = 0.04). Details regarding the significant correlations are presented in Table 5.

### 3.7. Regression Analysis

Multiple regression was run to predict body esteem from satisfaction with body image, use of TikTok, levels of stress, and loneliness. The multiple regression model statistically significantly predicted body esteem, F(4,422) = 93.704, *p* < 0.0001, adj. R^2^ = 0.46. All four variables added statistically significantly to the prediction, *p* < 0.05. 

Findings from the regression analysis for self-esteem indicate that levels of stress and depression, financial income and loneliness emerged as significant predictors for this variable. The multiple regression model indicated a small effect size for self-esteem, F(4,421) = 24.102, *p* < 0.0001, adj. R^2^ = 0.17. All three variables added statistically significantly to the prediction, *p* < 0.05. 

Multiple regression was conducted to predict loneliness from levels of stress, body image and self-esteem. The regression model indicated a small effect size for loneliness F(3,423) = 54.075, *p* < 0.0001, adj. R^2^ = 0.27. All three variables added statistically significantly to the prediction, *p* < 0.05. 

For all of these analyses, linearity was identified as assessed by partial regression plots and a plot of studentized residuals against the predicted values. Homoscedasticity was also evident, as assessed by visual inspection of a plot of studentized residuals versus unstandardized predicted values. There was no evidence of multicollinearity, as assessed by tolerance values greater than 0.1. The assumption of normality was met, as assessed by a Q-Q Plot. 

Regression coefficients and standard errors for these analyses are detailed in Table 6. 

## 4. Discussion

The present study investigated the presence of loneliness, self-esteem and body-esteem among students, and also identified the use of SNSs. Our study collected socio-demographic data together with self-rated satisfaction with physical and psychological health. The analysis of anthropometric data showed that 63% of students had a normal weight, and 80.6% exercised to lose weight or to prevent weight gain. We identified that students did not self-rate as being depressed but stressed, and the comparative analysis showed that men self-rated as being less depressed and less stressed, and in a better state of mental health, than women. Our results are congruent with Ljungberg et al. [24] and Iorga et al. [25], who conducted a study on students, and identified that freshmen are more depressed, whereas seniors are more stressed.

We identified that the most-used social networks were YouTube (98.8%), WhatsApp (98.4%), Facebook (93.2%) and Instagram (88.1%), and less than half of the students declared that they use Snapchat (48.5%) or TikTok (47.3%). The purpose of using SNSs was for socialization and entertainment, and less than one-fifth declared that they used social networks for discussion or solving academic tasks. Some studies from the literature showed that the most dangerous social media were Instagram, followed by Facebook and Twitter, due to the increasing number of posts edited and polished images [26].

Our study showed that a large majority of students (80.6%) dieted in order to maintain or reduce their weight. When comparing dieting and non-dieting respondents, we identified that there were significant differences between the two groups. Our results are in congruency with those obtained by Peternel and Sujoldžić [27]. The researchers found that students who were dieting reported significantly lower scores for self-esteem, lower levels for life satisfaction, lower body-image satisfaction, a higher rate of stress and a higher body mass index when compared to non-dieters. Tiggemann and Slater [28] showed that SNS use was correlated with internalization, body surveillance, negative body esteem, and dieting. 

We found that the higher the financial income, the higher the self-esteem. Additionally, the results show that the more income students had per month, the more likely they were to use YouTube more often. A study conducted by Peternel and Sujoldžić [27] confirmed that female respondents with low socioeconomic status were more prone to engage in dieting. 

We found that participants who engaged in physical activity in order to lose weight or to prevent weight gain obtained a lower score for BESSA than students who declared that they did not undertake physical activity for the mentioned purpose. 

The results proved that respondents had a higher level of body esteem with no gender differences. Our results are in opposition to those identified among female students but at a younger age. For example, Perloff [29] showed that SNS use can be detrimental to female teenagers’ body image because SNSs increase the negative impact of social media comparison more than traditional media. Some other studies identified that appearance comparisons on Facebook have a strongly negative impact on body esteem, but only on European girls and not in Asian girls. Therefore, many studies concluded that, at a younger age, the impact of images provided by SNSs will lead to comparisons, resulting in lower body esteem [30,31].

Kim and Kim [32] identified that female subjects were more dissatisfied with their body shape compared to men. Furthermore, the authors showed that men had higher levels of self-esteem. Moreover, a negative significant relation between BMI and self-esteem in women showed that the higher the rates of self-esteem among female respondents, the lower their BMI.

We identified that RSES analysis showed that the self-esteem total score was significantly higher in men than in women. Additionally, the results showed that there were significant differences in the RSES scores in the variable that refers to the self-assessment of satisfaction with body image; that is, those who estimated lower satisfaction with their own body obtained a lower score of self-esteem. Our findings are congruent with previous findings focusing on young SMS users. Social media use generates anxiety in adult women [33,34,35], and has been proven to have negative effects on body image outcomes, such as body esteem [11,14], body dissatisfaction [13] and body image [8].

Almost half of the students (45.2%, N = 1933) proved to have a moderate to high level of loneliness. As expected, we found that young adults who were in a relationship obtained a lower level of loneliness than single people.

Age was found to be an important factor. Our study showed that the younger the user, the higher the scores for loneliness and depression, and the greater the number of hours spent on SNSs. 

A study by Donnelly and Kuss [36] demonstrated that Instagram use and SNS addiction (where SNS addiction was defined by Schou Andreassen and Pallesen as “being overly concerned about SNSs, to be driven by a strong motivation to log on to or use SNSs, and to devote so much time and effort to SNSs that it impairs other social activities, studies/job, interpersonal relationships, and/or psychological health and well-being”) were significant predictors of depression [37]. The authors found no such relationship between Facebook, Twitter or Snapchat and SNS addiction or depression [36].

The analysis of the present data showed that, on average, students spent 3.38 ± 0.80 h per day using SNSs. The results are congruent with those presented in different articles. For example, Sampasa-Kanyinga and Lewis [38] showed that more than a quarter of the investigated students reported using SNSs for more than two hours every day. Their study identified that a daily use of more than two hours was correlated with a poor self-rating of mental health, high levels of psychological distress, and even suicidal ideation. The authors also showed that students with poor mental health were found to be more frequent users of SNS. Health status was found to be one of the most important predictors of loneliness, followed by social connectedness and, with much lower weights, lifestyle and socio-demographic factors [39]. 

Our results showed a significant correlation between the number of hours per day of using SNS and loneliness. The results presented by the scientific literature are contradictory. Burke et al. [40] and Krasnova et al. [41] identified that there was a significant correlation between SNS and loneliness, whereas Aparicio-Martinez [42] showed that there was no significant correlation between loneliness and the use of checking platforms. However, Gong et al. [17] conducted a study on medical students in China and identified that more than half (53.42%) of the participants used SNSs for at least one hour per day. The authors also proved that SNS addiction was positively related to depression, both directly and indirectly.

The study identified that, the more time the students spent on TikTok or Snapchat, the greater their body esteem level. Moreover, the use of Snapchat was strongly correlated with the RSES score, so the more the students spent time on Snapchat, the higher their self-esteem. The results are congruent with other studies. For example, in a literature review that analyzed twenty research studies on the relationship between body image and SNS use, Holland and Tiggemann [43] demonstrated that the overall time spent on SNSs is related to indices of body image in some studies; however, a large number of studies did not identify a negative impact of the time spent on SNSs on body-image satisfaction.

Al-Saggaf and Nielsen [44] concluded that more people who felt “lonely” on Facebook disclosed Personal Information, Relationship Information and Address compared to people who felt “connected”. The higher the student’s body mass index, the lower the body esteem score. Weight status was found to be negatively correlated with the use of TikTok.

The strength of the present research is due to its number of respondents; thus, the results generated by the study can be generalized for the population of young SNS users, especially medical students. The weak point of the research may be related to the unbalanced number of female and male respondents, and the comparative results must be considered with caution. The higher number of female students enrolled in medical studies is a general limitation presented by all studies conducted on medical students worldwide. Moreover, the results mirror the student population (especially medical students), so they must be considered to not be generalizable. Future research should focus on the difference between populations of young users with and without academic backgrounds. 

### Reflections and Planning

The use of SNSs is common among the young generation. The purposes for doing so are diverse, such as socializing, solving academic tasks, or even using them for medical or psychological issues. The authors suggest that the positive effects of SNS use are strongly related to the absence of addiction and psychological problems; otherwise, the more frequent the use, the stronger the vicious circle. The increasing number of hours invested in the use of SNSs is strongly related to some psychological problems, but it is sometimes difficult to clearly identify the process—does the number of hours determine the dissatisfaction with body image, or does the low satisfaction with the body image determine the self-isolation, loneliness and motivation to increase time spent on SNSs?

Practical implications for body image intervention programs, also targeting the decrease in loneliness and increasing self-esteem among teenagers, can be drawn from the findings of the present study.

## 5. Conclusions

The results of the present research proved the relationship between the use of SNSs and the presence of loneliness, self-esteem and body-esteem, with gender differences. In clinical settings, the use of SNSs should not be neglected due to their effective ability to reach patients and to provide medical and psychological intervention. In academic settings, psychologists and counselors must be aware of the impact of SNS use on psychological health, and provide preventive measures in order to decrease the risks of negative impacts. Due to the fact that age, gender, financial status and time spent on SNSs were found to be important factors, psychological and clinical interventions must be targeted on these aspects. Moreover, verifying addiction to SNSs may improve the psychological interventions among young SNSs users.

## Figures and Tables

**Table 1:** Socio-demographic and anthropometric data.

Socio-Demographic and Anthropometric Characteristics	M ± S.D and % ^1^
Marital status	
Single	200 (46.8%)
In a relationship	216 (50.6%)
Married	11 (2.6%)
Accommodation during the academic year	
Rent apartment	224 (52.5%)
Student dorm	96 (22.5%)
Parental home (with parents)	84 (19.7%)
with family (partner/husband/children)	22 (5.2%)
Co-habitants	
Single	87 (20.4%)
Colleagues/friends	211 (49.4%)
Partner	54 (12.6%)
Parents	75 (17.6%)
Average monthly financial income	
20–100 €	108 (25.3%)
100–200 €	148 (34.7%)
200–400 €	107 (25.1%)
>400 €	62 (14.5%)
Weight	64.12 ± 13.76
male	80.74 ± 14.83
female	60.58 ± 10.58
Body Mass Index	22.72 ± 3.94
male	25.32 ± 4.33
female	22.17 ± 3.62
Nutritional status (WHO)	
Underweight	48 (11.2%)
Normal weight	271 (63.5%)
Overweight	86 (20.1%)
Obese class I	19 (4.4%)
Obese class II	3 (0.7%)
Self-assessment of weight status	
Underweight	34 (8.0%)
Almost at a suitable weight	280 (65.6%)
Slightly overweight	93 (21.8%)
Very overweight	20 (4.7%)

^1^ Means and standard deviations (M ± D), frequency and percentages (%).

**Table 2:** Self-rated items regarding satisfaction with health-related status ^1^.

How Satisfied Are You with Your	1	2	3	4	5	M ± SD
physical condition	19, (4.4)	73, (17.1)	154, (36.1)	156, (36.5)	25, (5.9)	3.22 ± 0.94
mental state	33, (7.7)	69, (16.2)	127, (29.7)	165, (38.6)	33, (7.7)	3.22 ± 1.05
social relationships	17, (4.0)	45, (10.5)	104, (24.4)	191, (44.7)	70, (16.4)	3.59 ± 1.01
emotional support received from family	20, (4.7)	30, (7.0)	75, (17.6)	127, (29.7)	175, (41.1)	3.95 ± 1.13
emotional support received from your friends	11, (2.6)	24, (5.6)	105, (24.6)	187, (43.8)	100, (23.4)	3.80 ± 0.94

^1^ Number of answers (N) and percentage (%), means and standard deviations (M ± SD).

**Table 3:** Gender differences for the BESSA Scale ^1^.

Subscales	Men	Women
BES-Appearance	34.53 ± 8.62	33.23 ± 8.53
BES-Weight	26.52 ± 7.32	25.79 ± 8.11
BES-Attribution	22.54 ± 3.82	23.26 + 3.13

^1^ Means and standard deviations (M ± D).

**Table 4:** Results for the ULS-8 short form scale.

Loneliness Scale	N (%) ^1^
Low levels of loneliness	141 (33.0%)
Normal to moderate loneliness	193 (45.2%)
Moderate to high loneliness	80 (18.7%)
High levels of loneliness	13 (3.0%)

^1^ Number of answers (N) and percentage (%).

**Table 5:** Correlation analysis results.

Items	BESAA	RSES	USL-8
Satisfaction with the body image	r = 0.551, *p* = 0.000 **	r = 0.145, *p* = 0.003 **	r = −0.179, *p* = 0.000 **
Feeling depressed	r = −0.364, *p* = 0.000 **	r = −0.366, *p* = 0.001 **	r = 0.523, *p* = 0.000 **
Feeling stressed	r = −0.355, *p* = 0.000 **	r = −0.323, *p* = 0.000 **	r = 0.400, *p* = 0.000 **
What is the number of hours you spend on social media or messaging sites, such as Facebook, Twitter, and WhatsApp?	r = −0.102, *p* = 0.035 *	r = −0.117, *p* = 0.015 *	no correlation
RSES	r = 0.232, *p* = 0.000 **	1	r = −0.409, *p* = 0.030 **
ULS-8	r = −0.366, *p* = 0.000 **	r = −0.409, *p* = 0.030 **	1

* *p* < 0.05; ** *p* < 0.001.

**Table 6:** Regression coefficients and standard errors.

Independent Variables			95% CI for B		SE B			Collinearity Statistics		
		B	LL	UL		β	R ^2^	∆R ^2^	TI	VIF
BESAA	Model						0.47	0.46		
	Constant	54.599	44.778	64.419	4.996					
	How satisfied are you with your body image?	24.262	20.871	27.653	1.725	0.512			0.946	1.057
	How stressed do you feel?	−2.909	−4.101	−1.716	0.607	−0.186			0.837	1.194
	ULS-8	−0.813	−1.112	−0.515	0.152	−0.209			0.826	1.211
	Use of TikTok	4.174	1.680	6.668	1.269	0.117			0.994	1.006
RSES	Model						0.17	0.16		
	Constant	24.086	23.207	24.965	0.447					
	How stressed do you feel?	−0.284	−0.522	−0.045	0.121	−0.130			0.630	1.587
	ULS-8	−0.133	−0.189	−0.077	0.028	−0.245			0.711	1.407
	How depressed do you feel?	−0.291	−0.550	−0.032	0.132	−0.133			0.534	1.873
ULS-8	Model						0.27	0.27		
	Constant	27.140	22.975	31.305	2.119					
	How stressed do you feel?	0.895	0.533	1.257	0.184	0.222			0.816	1.226
	RSES	−0.439	−0.598	−0.280	0.081	−0.238			0.890	1.124
	BESAA	−0.067	−0.089	−0.044	0.011	−0.260			0.852	1.174

Model = “Enter” method in SPSS Statistics. B = unstandardized regression coefficient; CI = confidence interval; LL = lower limit; UL = upper limit; SE B = standard error of the coefficient; β = standardized coefficient; R^2^ = coefficient of determination; ∆R ^2^ = adjusted R ^2^; TI = tolerance statistic; VIF = variance inflation factor.

## Data Availability

The data presented in this study are available on request from the corresponding author.
